# Use of Ferritin Expression, Regulated by Neural Cell-Specific Promoters in Human Adipose Tissue-Derived Mesenchymal Stem Cells, to Monitor Differentiation with Magnetic Resonance Imaging In Vitro

**DOI:** 10.1371/journal.pone.0132480

**Published:** 2015-07-15

**Authors:** Chengang Song, Jiachuan Wang, Cuiping Mo, Shuhua Mu, Xiaogang Jiang, Xiaoyun Li, Shizhen Zhong, Zhenfu Zhao, Guangqian Zhou

**Affiliations:** 1 Department of Anatomy, Institute of Clinical Anatomy, Southern Medical University, Guangzhou, China; 2 School of Medicine, Shenzhen University, Shenzhen, China; 3 Department of Pathology, Shenzhen Affiliated Hospital, Guangzhou University of Traditional Chinese Medicine, Shenzhen, China; 4 Department of Pathology, Southern Medical University, Guangzhou, China; 5 Guangdong Provincial Key Lab of Biotechnology for Plant Development, College of Life Sciences, South China Normal University, Guangzhou, China; Shenzhen Institutes of Advanced Technology, CHINA

## Abstract

The purpose of this study was to establish a method for monitoring the neural differentiation of stem cells using ferritin transgene expression, under the control of a neural-differentiation-inducible promoter, and magnetic resonance imaging (MRI). Human adipose tissue-derived mesenchymal stem cells (hADMSCs) were transduced with a lentivirus containing the human ferritin heavy chain 1 (FTH1) gene coupled to one of three neural cell-specific promoters: human synapsin 1 promoter (SYN1p, for neurons), human glial fibrillary acidic protein promoter (GFAPp, for astrocytes), and human myelin basic protein promoter (MBPp, for oligodendrocytes). Three groups of neural-differentiation-inducible ferritin-expressing (NDIFE) hADMSCs were established: SYN1p-FTH1, GFAPp-FTH1, and MBPp-FTH1. The proliferation rate of the NDIFE hADMSCs was evaluated using a Cell Counting Kit-8 assay. Ferritin expression was assessed with western blotting and immunofluorescent staining before and after the induction of differentiation in NDIFE hADMSCs. The intracellular iron content was measured with Prussian blue iron staining and inductively coupled plasma mass spectrometry. R2 relaxation rates were measured with MRI in vitro. The proliferation rates of control and NDIFE hADMSCs did not differ significantly (*P* > 0.05). SYN1p-FTH1, GFAPp-FTH1, and MBPp-FTH1 hADMSCs expressed specific markers of neurons, astrocytes, and oligodendrocytes, respectively, after neural differentiation. Neural differentiation increased ferritin expression twofold, the intracellular iron content threefold, and the R2 relaxation rate two- to threefold in NDIFE hADMSCs, resulting in notable hypointensity in T2-weighted images (*P* < 0.05). These results were cross-validated. Thus, a link between neural differentiation and MRI signals (R2 relaxation rate) was established in hADMSCs. The use of MRI and neural-differentiation-inducible ferritin expression is a viable method for monitoring the neural differentiation of hADMSCs.

## Introduction

Most neurological disorders are caused by the loss of neurons or glial cells in the brain or spinal cord. Current therapies for these disorders are unable to replace damaged or lost neural cells. However, cell-based therapy offers the possibility of enhancing tissue repair and functional recovery in neurological disorders. Mesenchymal stem cells (MSCs) derived from bone marrow, umbilical cord blood, or adipose tissue are a promising cell source for cell-based therapies. MSCs have been used in several regenerative methods in animal models or patients with neurological diseases [1–4] and have been shown to enhance neurological recovery. Histological assays have confirmed that MSCs can differentiate along the neuronal lineage in vitro and in vivo [5–7]. Nevertheless, the fate of transplanted MSCs in live animals is still poorly understood. Thus, a noninvasive, real-time, sensitive, and clinically applicable method for tracking transplanted MSCs and monitoring their behavior in live animals would be useful.

Magnetic resonance imaging (MRI) is a suitable modality for the evaluation of stem cell therapy because of its excellent resolution and tissue contrast. MRI is commonly used for the noninvasive serial imaging of transplanted MSCs [8]. Many studies that have tracked transplanted MSCs in vivo have used superparamagnetic iron oxide (SPIO) particles as the MRI contrast agent [9]. However, the MRI signal hypointensity generated by these particles does not reflect the actual cell number because the iron oxide nanoparticles are diluted with each cell division. In addition, particles released from dead cells can be phagocytosed by host cells. Consequently, cell-labeling methods using SPIO particles are not suitable for the long-term monitoring of stem cell engraftment.

Genetic modification of cells in vitro to induce the expression of a reporter gene encoding an MRI-detectable probe is a novel approach to transplanted-cell imaging. The use of reporter genes for MRI-based cell tracking is advantageous for the longitudinal monitoring of cell transplants because gene expression correlates much more tightly than particle retention with cell viability and because transgene-based reporters are much less susceptible to signal loss through cell division. Ferritin is a ubiquitous intracellular protein that stores iron in a nontoxic form and releases it in a controlled manner. Ferritin overexpression for MRI visualization of transplanted cells has been assessed in several studies [10–12], and the results suggest that ferritin can be used to track the survival, growth, and migration of transplanted stem cells. Nonetheless, the use of ferritin overexpression to monitor the neural differentiation of transplanted stem cells noninvasively has not been investigated. In the present study, we developed an MRI imaging technique for assessing the neural differentiation of ferritin-tagged transplanted cells. Neural cell-specific promoters were used to regulate ferritin expression, and the ability to monitor cell differentiation in live cells was assessed.

## Materials and Methods

### Construction of neural-differentiation-inducible ferritin-expressing (NDIFE) recombinant lentiviral vectors

The open reading frames (ORFs) of human ferritin heavy chain 1 (FTH1) and three neural cell-specific (neural-differentiation-inducible) promoter sequences (human neuron synapsin I promoter [SYN1p], human astrocyte glial fibrillary acidic protein promoter [GFAPp] [13], and human oligodendrocyte myelin basic protein promoter [MBPp] [14]) (Table 1) were designed to contain the restriction enzyme sites needed for cloning. The ORFs were amplified by PCR using the templates and primers listed in S1 Table.

**Table 1:** Target sequences used in the present study.

Target sequence	GenBank accession number	Reference
FTH1 ORF	BC015156 (59–610)^a^	[10]
SYN1p	M55301.1 (1889–2357)	[13]
GFAPp	M67446.1 (1–2210)	[13]
MBPp	NC_000018.10 (115904–116653)	[14]

^a^Position according to the GenBank sequence.

FTH1: ferritin heavy chain 1, ORF: open reading frame, SYN1: synapsin I, GFAP: glial fibrillary acidic protein, MBP: myelin basic protein.

**Fig 1:**
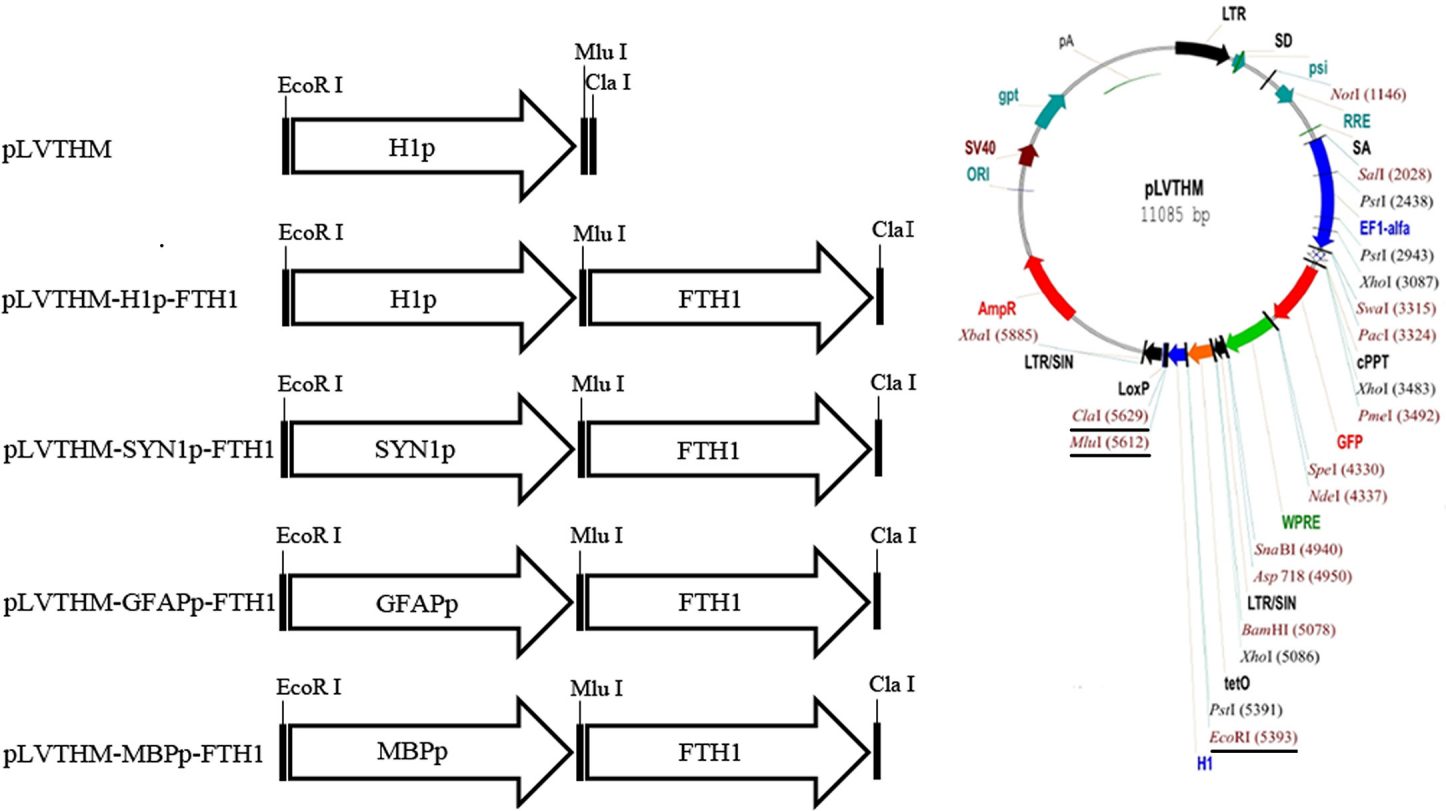
Outline of lentiviral vector construction. Ferritin-expressing lentiviral vectors were constructed in two steps. First, the *FTH1* sequence was cloned into the pLVTHM vector between the MluI and ClaI sites, downstream of the H1p promoter, generating the pLVTHM-H1p-FTH1 plasmid vector. Second, one of three neural cell-specific promoter sequences (SYN1p, GFAPp, or MBPp) was cloned into the pLVTHM-H1p-hFTH1 vector between the EcoRI and MluI sites, replacing the H1p sequence, to generate three NDIFE vectors: pLVTHM-SYN1p-FTH1, pLVTHM-GFAPp-FTH1, and pLVTHM-MBPp-FTH1. FTH1: ferritin heavy chain, SYN1: synapsin I, GFAP: glial fibrillary acidic protein, MBP: myelin basic protein.

The FTH1 ORF sequence was cloned into the pLVTHM vector (Addgene, cat. # 12247, Cambridge MA, USA) between the MluI and ClaI sites, downstream of the H1 promoter (H1p), generating the pLVTHM-H1p-FTH1 plasmid vector. The three neural-differentiation-inducible promoter sequences were then cloned individually into pLVTHM-H1p-FTH1 upstream of the FTH1 sequence, replacing the H1p sequence. Three NDIFE vectors were thus generated: pLVTHM-SYN1p-FTH1, pLVTHM-GFAPp-FTH1, and pLVTHM-MBPp-FTH1 (Fig 1). The structures of the new plasmids were verified with DNA sequencing.

### Production of NDIFE lentivirus particles

Sixth-passage HEK293T cells (cat. # C0002; Biowit Technologies, Shenzhen, China; obtained directly from the company) were cultured in 10-cm culture dishes in high-glucose Dulbecco’s modified Eagle’s medium (DMEM) supplemented with 10% fetal bovine serum (FBS) until they reached 70–80% confluence. The three NDIFE vectors were individually cotransfected with the pMD2.G envelope plasmid (cat. # 12259; Addgene) and the psPAX2 packaging plasmid (cat. # 12260; Addgene) into HEK-293T cells using the Calcium Phosphate Cell Transfection Kit (cat. # C0037; Beyotime, Nantong, China). Virus-containing culture supernatants were collected, combined, centrifuged at 500 × *g* for 5 min, and clarified with a membrane filter (0.45-μm pore size; cat. #SLHA02510; Millipore, Bedford, MA, USA) to remove cell debris. For concentration by ultrafiltration, the filtrate was placed in an Amicon Ultra-15 100K NMWL (Nominal Molecular Weight Limit) Centrifugal Filter device (cat. # UFC910008; Millipore) and centrifuged at 5000 × *g* for 20 min at 4°C. Viral titers (transducing units/mL) were determined by counting GFP-positive HEK293T cells transfected with serial dilutions of the concentrated viral-culture supernatant. The titers of the three NDIFE lentiviruses were in the range of 10^7^–10^8^ transduction units per milliliter. The lentiviruses were dispensed into multiple tubes and stored at −80°C.

### Human adipose tissue-derived mesenchymal stem cells (hADMSCs): culture and lentiviral transduction

Second-passage hADMSCs (cat. # C0013; Biowit Technologies, Shenzhen, China; obtained directly from the company) were seeded, cultured, and propagated in complete medium (high-glucose DMEM supplemented with 10% FBS, 10 mM *N*-2-hydroxyethylpiperazine-*N*-2-ethane sulfonic acid, 2 mM l-glutamine, 100 U/mL penicillin, and 100 μg/mL streptomycin) in a humidified atmosphere containing 5% CO_2_ at 37°C.

For lentiviral transduction, fifth-passage hADMSCs were grown to 70–80% confluence and transduced with one of the three NDIFE lentiviruses at a multiplicity of infection of 100 in the presence of 6 μg/mL protamine sulfate (cat. # P4020; Sigma-Aldrich, Shanghai, China) for 24 h to generate three groups of stably transduced NDIFE hADMSCs: SYN1p-FTH1, GFAPp-FTH1, and MBPp-FTH1 hADMSCs. The transduction efficiency was evaluated by assessing the expression of GFP (expected to be present in approximately 70% of the cells). Stably transduced NDIFE hADMSCs were maintained and propagated for subsequent experiments.

### Cell proliferation assay

HADMSC proliferation was evaluated using the Cell Counting Kit-8 assay (cat. # C0037; Beyotime). HADMSCs were plated at a density of 1500 cells/well and cultured in 96-well plates for 24, 48, 72, or 96 h. The absorbance at 450 nm was then measured using an Epoch Multi-Volume Spectrophotometer System (BioTek, Winooski, VT, USA). All experiments were independently performed in triplicate.

### Neural differentiation of NDIFE hADMSCs

SYN1p-FTH1, GFAPp-FTH1, and MBPp-FTH1 NDIFE hADMSCs were induced to differentiate into neurons, astrocytes, and oligodendrocytes, respectively, using protocols described previously [15–18]. The following neurally differentiated NDIFE (ND-NDIFE) hADMSCs were thereby generated: SYN1p-FTH1-N, GFAPp-FTH1-A, and MBPp-FTH1-O hADMSCs. In brief, NDIFE hADMSCs were plated in complete medium at 8000 cells/cm^2^ on tissue culture coverslips (cat. # 01022014; WHB, Shanghai, China) in 24-well plates. The differentiation induction procedures are described in S2 Table. The expression of neural cell-specific markers in the hADMSCs was assessed with immunofluorescence staining.

### Immunofluorescence staining

HADMSCs cultured on tissue culture coverslips were fixed in 4% paraformaldehyde for 15 min, washed in phosphate-buffered saline (PBS), permeabilized with 0.1% Triton X-100 in PBS for 15 min, and blocked with blocking buffer (PBS containing 5% bovine serum albumin) for 1 h at room temperature. The cells were then incubated with primary antibodies overnight at 4°C. For immunofluorescence staining of neural cell-specific markers before and after neural differentiation, we used the following primary antibodies: a rabbit polyclonal anti-neuron-specific enolase (NSE) antibody (1:500, cat. # ab64721; Abcam, Cambridge, MA, USA) to assess neuronal differentiation, a mouse monoclonal anti-GFAP antibody (1:100, cat. # ab4648; Abcam) to assess astrocytic differentiation, and a mouse monoclonal anti-MBP antibody (1:1000, cat. # ab24567; Abcam) to evaluate oligodendrocytic differentiation. For immunofluorescence staining of ferritin, the primary antibody was a rabbit polyclonal anti-hFTH1 antibody (1:1000, cat. # ab65080; Abcam). The cells were washed in PBS and incubated with the appropriate DyLight 649-conjugated goat anti-rabbit IgG secondary antibody (1:3000; cat. # 042-05-18-06 or 042-05-15-06; KPL, Gaithersburg, MD, USA) in the dark for 1 h at room temperature. Subsequently, cell nuclei were counterstained with 4′,6-diamidino-2-phenylindole (DAPI) Fluoromount-G (cat. # 0100–20; SouthernBiotech, Birmingham, AL, USA) while the coverslips were mounted. Images were acquired under a fluorescence microscope (BX51; Olympus, Tokyo, Japan). The control coverslips were treated identically, but the primary antibody was omitted.

### Iron loading

To assess the intracellular iron content and MRI R2 relaxation rates in hADMSCs in vitro, we maintained each group of hADMSCs in the relevant medium with 200 μM ferric ammonium citrate (FAC) (cat. # 09713; Sigma-Aldrich, Shanghai, China) for 72 h before testing.

### Western blot analysis

HADMSCs (3 × 10^5^) in 6-well plates were harvested and lysed in RIPA Lysis and Extraction Buffer (cat. # 89900; Pierce, Rockford, lL, USA) containing the Halt Protease Inhibitor Cocktail (cat. # 78430; Pierce) and Halt Phosphatase Inhibitor Cocktail (cat. # 78420; Pierce). The total protein concentration in the samples was measured with a bicinchoninic acid protein assay kit (cat. # 23225; Pierce). We then added 4× loading buffer to the samples and boiled them for 5 min. Equal amounts of protein (30 μg/lane) from each group of hADMSCs were separated with 15% sodium dodecyl sulfate-polyacrylamide gel electrophoresis and transferred onto polyvinylidene difluoride membranes (Millipore). To detect human ferritin, we blocked the membranes with blocking buffer (Tris-buffered saline with 0.1% Tween-20 and 5% skim milk) at room temperature for 1 h and then incubated the membranes with a rabbit polyclonal anti-hFTH1 primary antibody (1:1000, cat. # 65080; Abcam) or a mouse monoclonal anti-β-actin antibody (1:5000, cat. # CW0096; CWBIO, Beijing, China) overnight at 4°C. The membranes were washed with TBS-T and incubated with an appropriate horseradish peroxidase-conjugated secondary antibody (1:5000, cat. # CW0102 or CW0103; CWBIO). The relative expression of ferritin in hADMSCs was analyzed semiquantitatively from the band intensity using the Quantity One software (Bio-Rad, Hercules, CA, USA), with normalization to human β-actin.

### Evaluation of the intracellular iron content

The average intracellular iron content per cell was quantified using inductively coupled plasma mass spectrometry (ICP-MS; Thermo X Series II) with cell count normalization. Each group of hADMSCs from a 10-cm culture dish was washed thoroughly with PBS to remove free FAC, trypsinized, harvested, and counted (to obtain 3 × 10^6^ cells/group). The cell pellets were resuspended in 50 μL of 68% (v/v) concentrated nitric acid (guaranteed reagent grade; JHD, Guangdong, China), digested at 90°C overnight, and diluted with 18-megaohm deionized water to 1000 μL.

In addition, intracellular iron accumulation and distribution in the hADMSCs were qualitatively assessed with Prussian blue iron staining. HADMSCs grown on coverslips were washed with PBS, fixed in 4% paraformaldehyde for 20 min, stained with a staining solution (2% potassium ferrocyanide and 3% HCl) for 30 min, and washed with PBS. Cell nuclei were counterstained with a nuclear fast red solution (0.1% nuclear fast red and 5% aluminum sulfate). The coverslips were mounted and examined under a microscope (BX51; Olympus).

### In vitro MRI

HADMSCs (5 × 10^6^/group) were washed with PBS to remove free FAC, trypsinized, harvested, and uniformly embedded into 600 μL of 1% agarose to prepare cell phantoms in 600-μL Eppendorf tubes for in vitro MRI. T2-weighted MRI was performed on a 3T MAGNETOM Trio MRI system (Siemens, Germany), and the T2 values were determined using a multi-echo spin echo T2-weighted sequence with the following parameters: TR, 4000 ms; eight echo settings of 13, 26, 39, 52, 65, 78, 91, and 104 ms; matrix, 64 × 128; field of view, 50 × 100 mm; and a horizontal slice with 2-mm slice thickness at the center of each Eppendorf tube.

### Statistical analysis

Quantitative data have been presented as the mean ± standard deviation (SD). To examine differences among the groups, one-way analysis of variance (ANOVA) was applied using the SPSS 13.0 software (SPSS Inc., Chicago, IL, USA). Differences were considered statistically significant at *P* < 0.05.

## Results

### Overexpression of ferritin does not affect hADMSC proliferation

**Fig 2:**
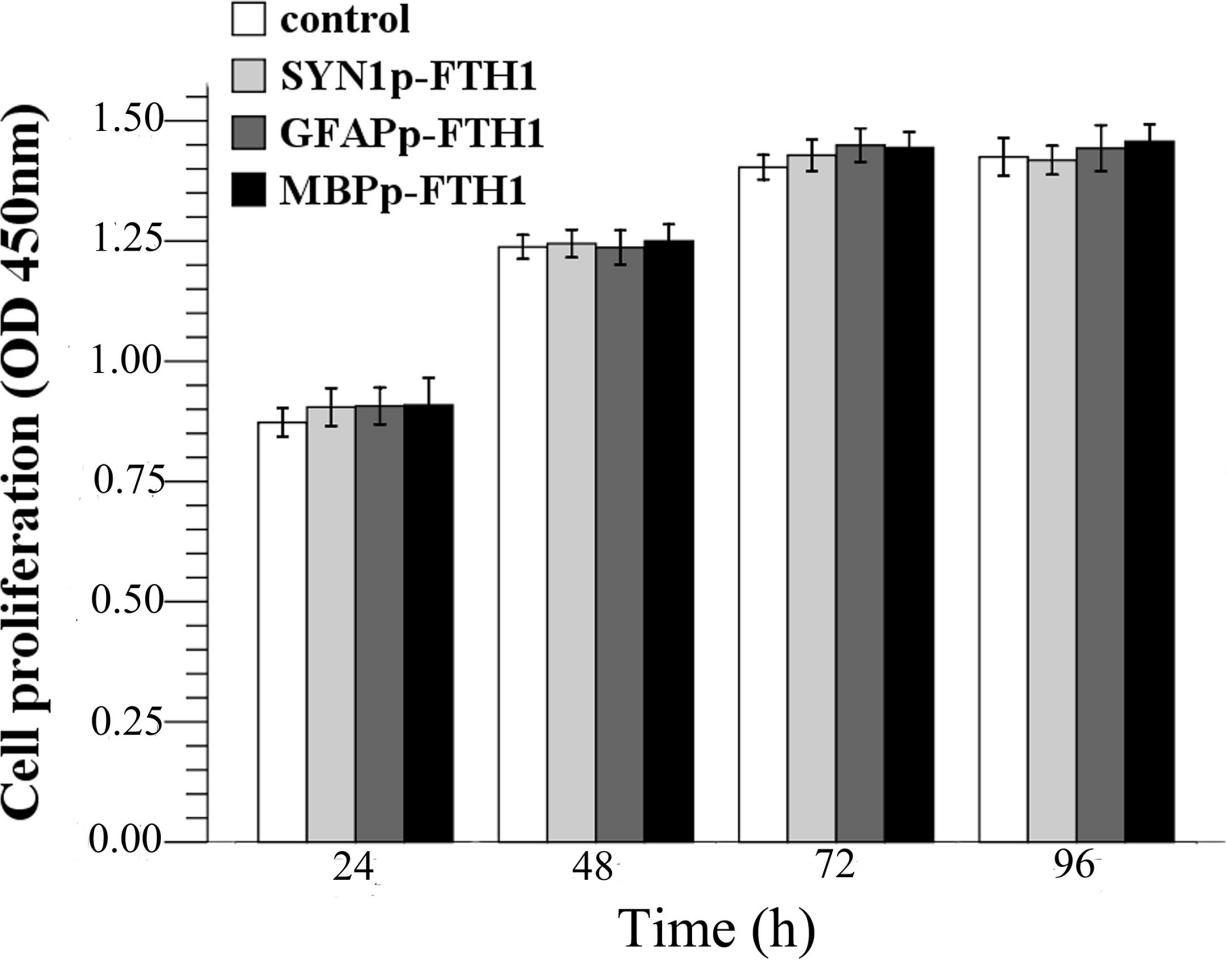
Effects of the ferritin transgene on human adipose tissue-derived mesenchymal stem cell (hADMSC) proliferation. There were no significant differences between the control group and the NDIFE hADMSC groups at each time point (one-way analysis of variance, *P* > 0.05). Error bars represent the mean ± standard deviation (n = 3). The ferritin transgene did not affect the proliferation rate of hADMSCs.

To test whether ferritin transgene overexpression inhibited hADMSC proliferation, cell proliferation was measured during a 96-h period (Fig 2). From 24 to 72 h, all cells underwent logarithmic growth and then entered a plateau phase from 72 h to 96 h. There were no statistically significant differences between the proliferation rates of the hADMSC groups (*P* > 0.05). The results suggested that ferritin overexpression did not inhibit hADMSC proliferation.

### Neural differentiation of NDIFE hADMSCs

**Fig 3:**
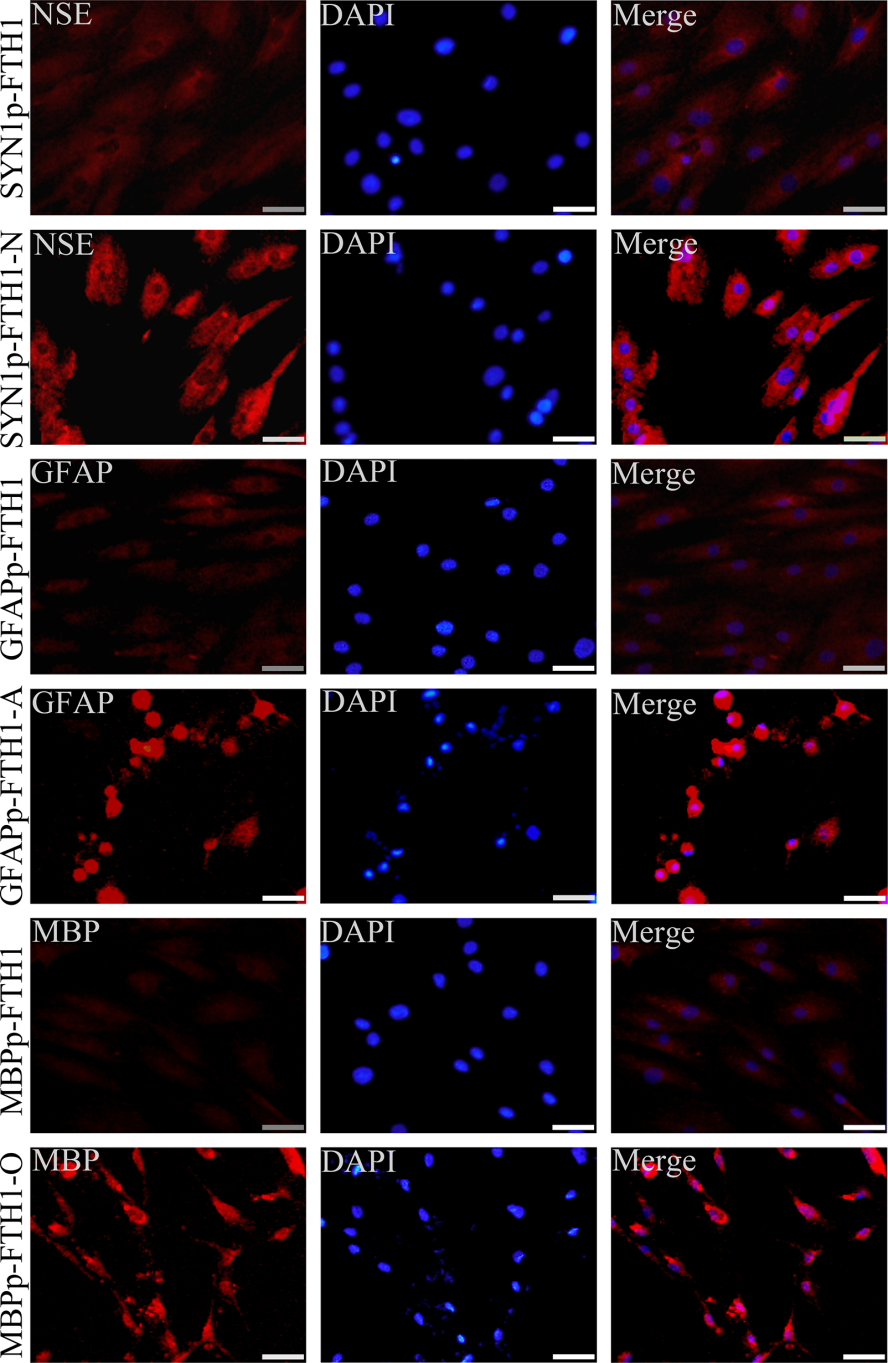
Expression of neural cell-specific markers in differentiating neural-differentiation-inducible ferritin-expressing human adipose tissue-derived mesenchymal stem cells. After neural differentiation, neural-differentiation-inducible ferritin-expressing (NDIFE) human adipose tissue-derived mesenchymal stem cells (hADMSCs) began to express neural cell-specific markers. The scale bar is 50 μm. SYN1: synapsin I, NSE: neuron-specific enolase, FTH1: ferritin heavy chain 1, GFAP: glial fibrillary acidic protein, MBP: myelin basic protein, DAPI: 4′,6-diamidino-2-phenylindole.

In NDIFE hADMSCs, the expression of neural cell-specific markers changed after neural differentiation (Fig 3). Untreated (undifferentiated) NDIFE hADMSCs were weakly positive for NSE, GFAP, and MBP expression. The SYN1p-FTH1, GFAPp-FTH1 and GFAPp-FTH1 hADMSCs strongly expressed the neuron-specific marker NSE, astrocyte-specific marker GFAP and oligodendrocyte-specific marker MBP after neuronal, astrocytic and oligodendrocytic differentiation respectively.

### Neural differentiation increases ferritin expression in NDIFE hADMSCs

**Fig 4:**
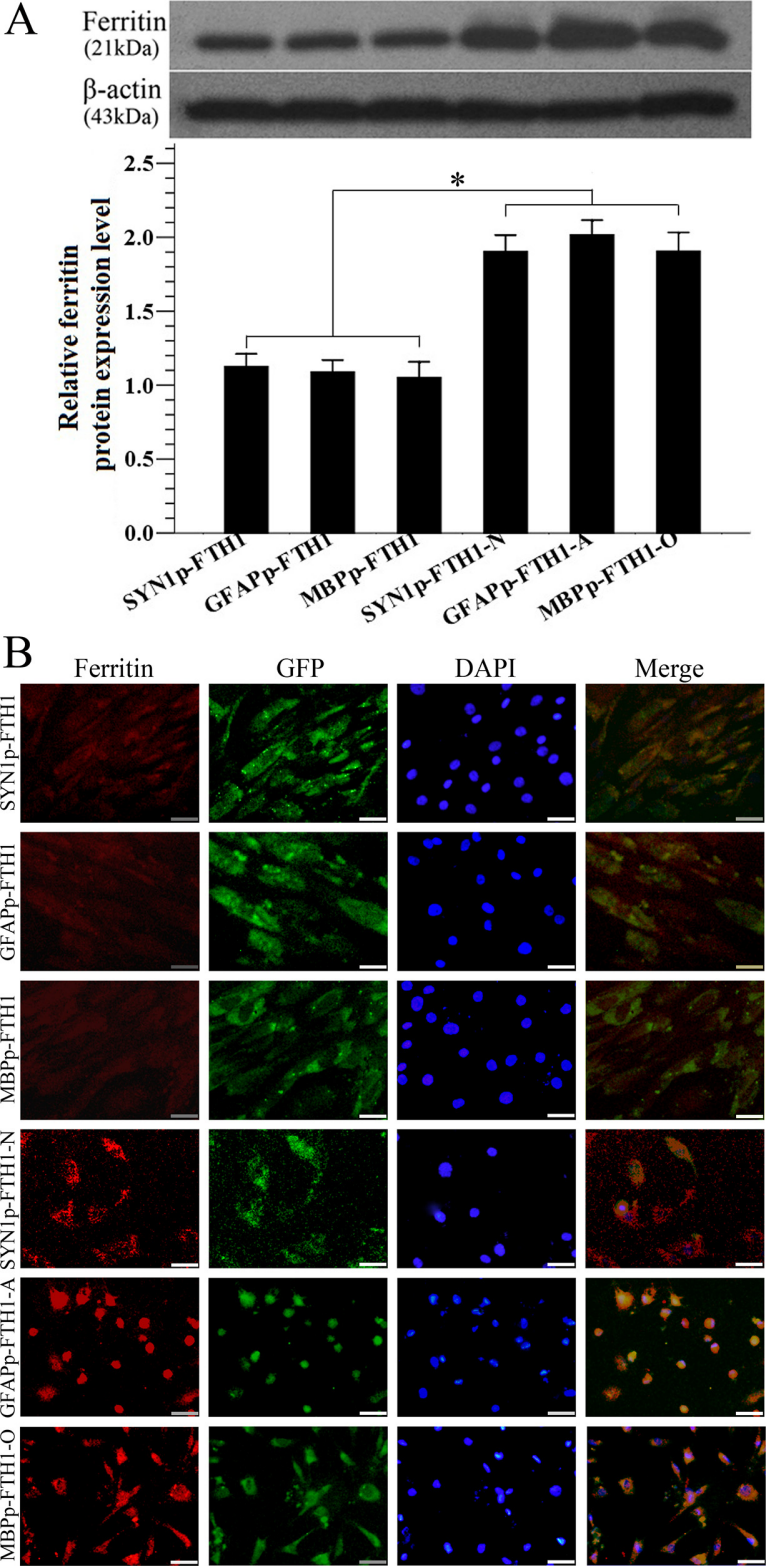
Neural differentiation increases ferritin expression in neural-differentiation-inducible ferritin-expressing human adipose tissue-derived mesenchymal stem cells. (A) Western blot analysis of ferritin expression in neural-differentiation-inducible ferritin-expressing (NDIFE) and neurally differentiated (ND)-NDIFE human adipose tissue-derived mesenchymal stem cells (hADMSCs). Ferritin expression was significantly higher in ND-NDIFE hADMSCs than in the corresponding control NDIFE hADMSCs. Error bars represent the mean ± standard deviation (n = 3); *P < 0.05. (B) A photomicrograph showing the immunofluorescent staining of ferritin in NDIFE and ND-NDIFE hADMSCs. Ferritin expression (red fluorescence) was significantly stronger in ND-NDIFE hADMSCs than in the corresponding control NDIFE hADMSCs. The scale bar is 50 μm. GFP: green fluorescent protein, DAPI: 4′,6-diamidino-2-phenylindole.

Western blotting was used to examine ferritin expression in hADMSCs. Ferritin levels in ND-NDIFE hADMSCs were ~twofold higher than those in the corresponding control NDIFE hADMSCs (*P* < 0.05; Fig 4A). Similar results were observed with immunofluorescence staining: ferritin expression (red fluorescence) was significantly stronger in ND-NDIFE hADMSCs than in the corresponding control NDIFE hADMSCs (Fig 4B). These results indicated that neural differentiation significantly increased the activity of neural-differentiation-inducible promoters and upregulated downstream ferritin gene expression in the NDIFE hADMSCs.

### Neural differentiation increases the intracellular iron concentration in NDIFE hADMSCs

**Fig 5:**
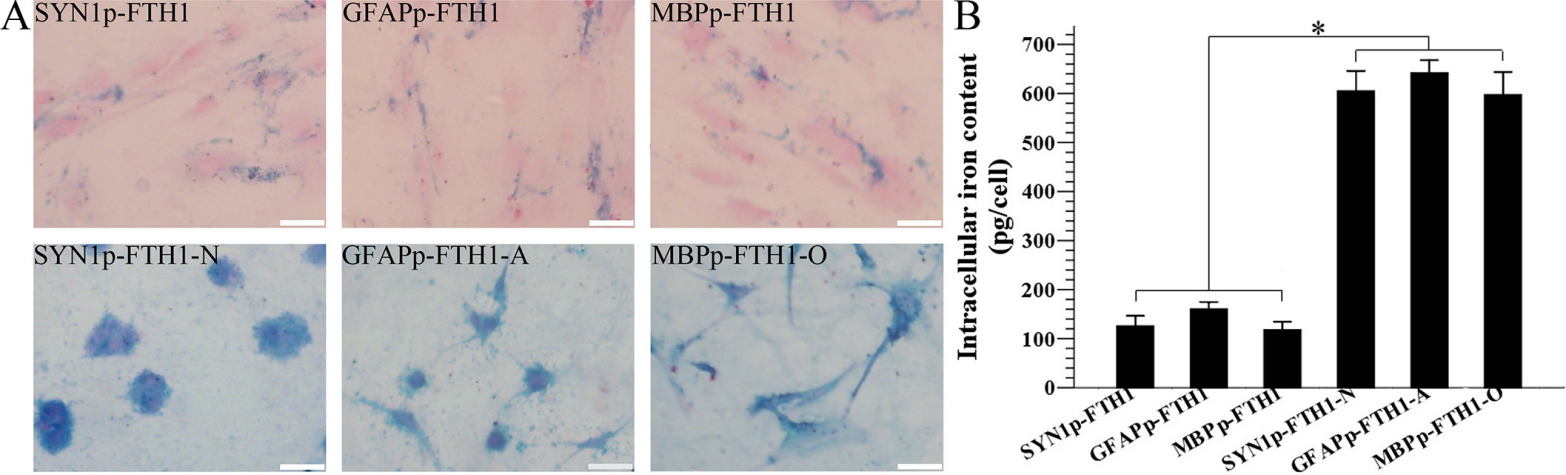
Differentiation increases the intracellular iron level in neural-differentiation-inducible ferritin-expressing human adipose tissue-derived mesenchymal stem cells. (A) A photomicrograph showing Prussian blue iron staining of intracellular iron in neural-differentiation-inducible ferritin-expressing (NDIFE) and neurally differentiated (ND)-NDIFE human adipose tissue-derived mesenchymal stem cells (hADMSCs). Many disperse cytoplasmic deposits (blue granules) of accumulated intracellular iron were observed in NDIFE hADMSCs, whereas large, dense cytoplasmic deposits appeared in the corresponding control ND-NDIFE hADMSCs. The scale bar is 50 μm. (B) Inductively coupled plasma mass spectrometry (ICP-MS) analysis of the intracellular iron content in NDIFE and ND-NDIFE hADMSCs. The intracellular iron content in ND-NDIFE hADMSCs was significantly higher than that in the corresponding undifferentiated NDIFE hADMSCs. Error bars represent the mean ± standard deviation (n = 3; *P < 0.05).

The effect of ferritin expression on the intracellular iron content was assayed with ICP-MS and Prussian blue staining. In the qualitative analysis, many disperse deposits (blue granules) of accumulated intracellular iron were present in the cytoplasm of NDIFE hADMSCs. After the NDIFE hADMSCs underwent neural differentiation, large, dense deposits of accumulated iron were observed throughout the cytoplasm. The results indicated that the intracellular iron content increased dramatically relative to the iron content in undifferentiated NDIFE hADMSCs (Fig 5A).

In the ICP-MS quantitative analysis, the intracellular iron content (pg/cell) was approximately threefold higher in the ND-NDIFE hADMSCs than in the corresponding undifferentiated NDIFE hADMSCs (*P* < 0.05; Fig 5B). Ferritin expression appeared to increase as a result of neural differentiation. The fact that the intracellular iron content increased notably suggested that the cells’ ability to internalize and store iron had improved.

### Neural differentiation increases the R2 relaxation rate in NDIFE hADMSCs in vitro

**Fig 6:**
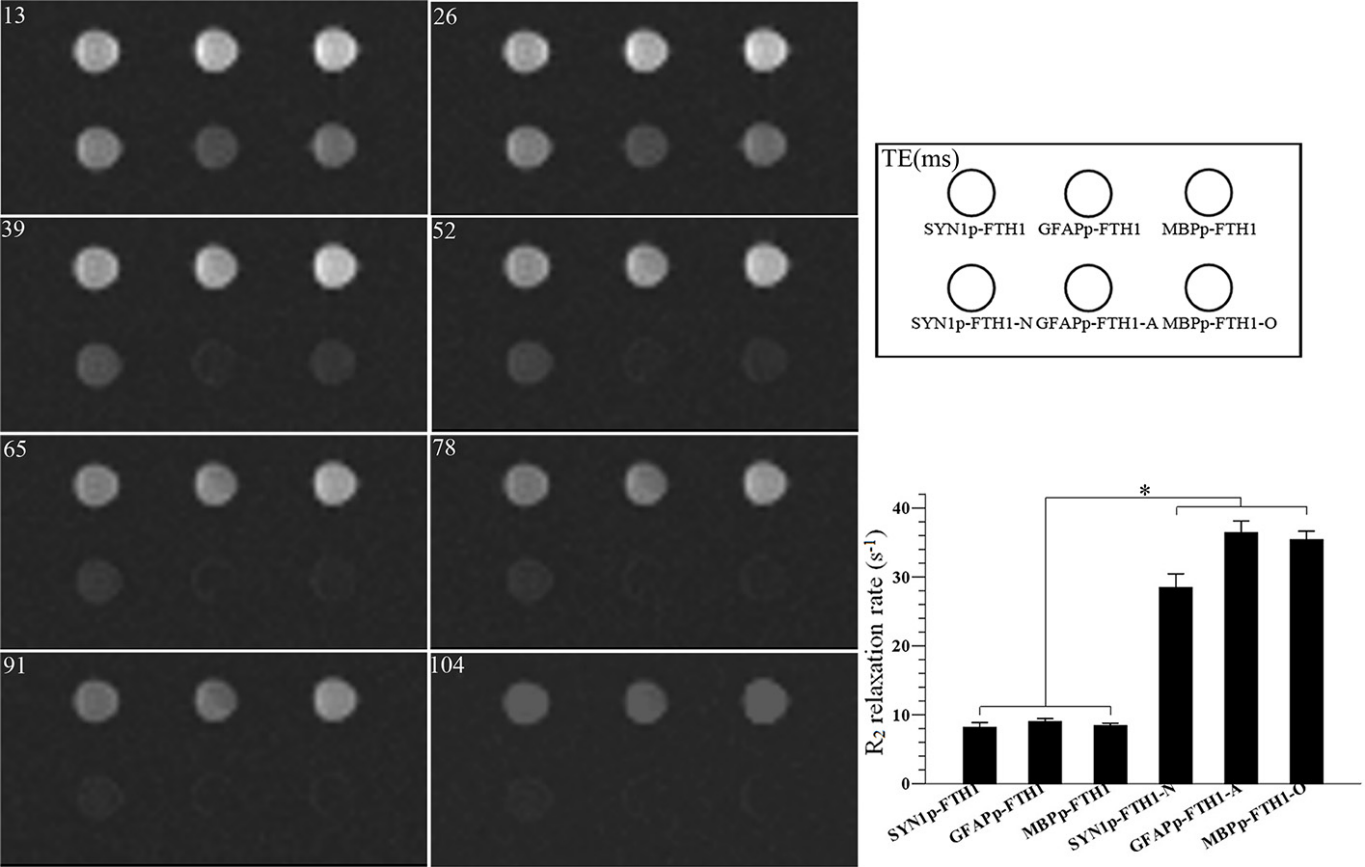
Differentiation increases the R2 relaxation rates of neural-differentiation-inducible ferritin-expressing human adipose tissue-derived mesenchymal stem cells. A T2-weighted image from magnetic resonance imaging (MRI) of agarose phantoms of neural-differentiation-inducible ferritin-expressing (NDIFE) and neurally differentiated (ND)-NDIFE human adipose tissue-derived mesenchymal stem cells (hADMSCs). The R2 relaxation rate of ND-NDIFE hADMSCs was significantly higher than that of the corresponding undifferentiated NDIFE hADMSCs, resulting in notable hypointensity in T2-weighted images of ND-NDIFE hADMSCs. Error bars represent the mean ± standard deviation (n = 4; *P < 0.05).

T2-weighted magnetic resonance images and R2 relaxation rates (1/T2) of hADMSC agarose phantoms were acquired on a 3.0-T MAGNETOM Trio scanner. The R2 relaxation rate in ND-NDIFE hADMSCs was approximately two- to threefold higher than that in the corresponding undifferentiated NDIFE hADMSCs; the difference resulted in notable hypointensity in the T2-weighted images of ND-NDIFE hADMSCs (*P* < 0.05; Fig 6). These data showed that MRI could detect in vitro the differences in ferritin expression resulting from the neural differentiation of NDIFE hADMSCs.

## Discussion

The objective of this study was to develop a method for monitoring neural differentiation of stem cells and lay a basis for monitoring their fates in live animals. MRI was used to measure ferritin, which was expressed under the control of a neural cell-specific promoter (neural-differentiation-inducible promoter). We hypothesized that neural differentiation would enhance the activity of the neural-differentiation-inducible promoter in NDIFE hADMSCs and that the expression of the downstream ferritin transgene would thus increase markedly, resulting in a strong increase in the intracellular iron content and the R2 relaxation rate. To test this hypothesis, we analyzed ferritin expression, the intracellular iron content, and T2-weighted images of NDIFE hADMSCs before and after their differentiation into neurons, astrocytes, or oligodendrocytes.

In this study, FTH1 overexpression did not inhibit hADMSC proliferation. These results are consistent with those of other MSC tracking studies using lentiviral ferritin transduction [11,19]. Our findings indicate that ferritin expressed from a lentiviral vector is a suitable MRI reporter for the labeling and tracking of stem cells.

Neural cell-specific promoters derived from neural cell-specific marker genes (e.g., neuronal SYN1, astrocytic GFAP, and oligodendrocytic MBP) drive target gene expression nearly exclusively in specific cell types [13,14]. In this study, neural differentiation significantly enhanced ferritin expression, resulting in a notable increase in the intracellular iron concentration and R2 relaxation rate (hypointensity in the T2-weighted magnetic resonance images) in NDIFE hADMSCs. This finding suggests that the baseline activities of the neural cell-specific promoters in NDIFE hADMSCs increase when NDIFE hADMSCs undergo neural differentiation. Many studies have shown that MSCs have a tendency to differentiate along the neural lineage and express neural stem cell markers, such as nestin, to some extent even before induction, suggesting that MSCs are already committed to neural differentiation [20,21]. In this study, the expression of the ferritin transgene, the intracellular iron content, and the MRI R2 relaxation rate were cross-validated successfully. Our findings are consistent with the idea that an increase in the R2 relaxation rate, i.e., a shortening of the T2 relaxation time (1/R2), is directly proportional to the iron concentration [22]. Thus, a neural-differentiation-inducible promoter can serve as a “regulatory valve” that controls ferritin expression in NDIFE hADMSCs before and after neural differentiation. Thus, a link between neural differentiation and MRI signals (R2 relaxation rate) in hADMSCs was established. To the best of our knowledge, this is the first study in which MRI was used to monitor the neural differentiation of hADMSCs in vitro.

In contrast to transduced ferritin, iron oxide nanoparticles are exogenous, and their intracellular concentration cannot be regulated by neural cell-specific promoters. Thus, the correlation between the neural differentiation of hADMSCs and the MRI signals from iron oxide nanoparticles is not coincidental. On the other hand, monitoring the neural differentiation status in vivo (as opposed to in vitro) might be complicated because the neural differentiation of transplanted cells is generally almost synchronous with migration in response to a lesion in living organisms. Further in vivo research using models of nervous system diseases is warranted.

In summary, ferritin, expressed under the control of a neural-differentiation-inducible promoter, is a suitable MRI marker for monitoring the neural differentiation of hADMSCs. Furthermore, the method might have other applications. For example, inducible (specific) promoters, acting as “regulatory valves” to control the expression of target genes, could be used to evaluate the biosafety profile of stem cells and monitor the malignant transformation of tumor cells.

## Supporting Information

S1 Table Templates and primers for PCR amplification. (DOC)

S2 Table Neural differentiation procedures for neural-differentiation-inducible ferritin-expressing (NDIFE) human adipose tissue-derived mesenchymal stem cells (hADMSCs). (DOC)

S3 Table The raw data of this study. Table S3-1. Raw data of Cell Counting Kit-8 assay. Table S3-2. Raw data of Western blot. Table S3-3. Raw data of inductively coupled plasma mass spectrometry. Table S3-4. Raw data of MRI in vitro.
(DOC)
