# Supplementary material for: Use of Ferritin Expression, Regulated by Neural Cell-Specific Promoters in Human Adipose Tissue-Derived Mesenchymal Stem Cells, to Monitor Differentiation with Magnetic Resonance Imaging In Vitro
Source: PLoS One. 2015 Jul 15;10(7):e0132480. doi: 10.1371/journal.pone.0132480 (PMC4503445; doi:10.1371/journal.pone.0132480)
Supplement: S1 Table — (DOC) [file pone.0132480.s001.doc]

**S1 Table. Templates and primers for PCR amplification.**

| Target sequence | Template | Primer sequence (5′→3′) | Size (bp) |
| --- | --- | --- | --- |
| hFTH1 ORF | K562 cells, total RNA | pLVTHM-hFTH1-Mlu I-F:  5′CGGACGCGTCACCATGACGACCGCGTCCACCTCGCAGGTGCGC3′  pLVTHM-hFTH1-Cla I-R:  5′CCCATCGATTCACTTGTCGTCGTCATCCTTGTAATCGCT-3′ | 552 |
| hSYN1p | pLV-hSyn-RFP  (Addgene 22909) | pLVTHM-hSYN1-EcoR I-F:  5′CCGGAATTCCTGCAGAGGGCCCTGCGTATGAGTG3′  pLVTHM-hSYN1-Mlu I-R:  5′CGGACGCGTCTGCGCTCTCAGGCACGACACGACT3′ | 469 |
| hGFAPp | U251 cells, genomic DNA | pLVTHM-hGFAP-EcoR I-F:  5′CCGGAATTCGAGCTCCCACCTCCCTCTCTGTGCTG3′  pLVTHM-hGFAPmutant-Mlu I-R:  5′CGGACGCGTGCGAGCAGCGGAGGTGATGCGTCTCC  TCTCCAACCTGCTCTGGCTCAGC3′ | 2210 |
| hMBPp | U251 cells, genomic DNA | pLVTHM-hMBP-Mfe I-F:  5′CCGCAATTGGGCCTCTTGTGGAATCTGGGATTGA3′  pLVTHM-hMBP-Mlu I-R:  5′CGGACGCGTGGAGCTCGTCGGACTCAGAGGGCCTGTCTT3′ | 750 |

Sources of cell lines and reagents: K562 cells (cat. # C0002; Biowit Technologies, Shenzhen, China; obtained directly from company); U251 cells (cat. # CX0319; Boster, Wuhan, China; obtained directly from company); primer synthesis and vector sequencing (Invitrogen, Shanghai, China); reagents for reverse transcription, PCR, digestion, cloning (Thermo Scientific, Shanghai, China; obtained directly from company).

FTH1: ferritin heavy chain, ORF: open reading frame, SYN1: synapsin I, GFAP: glial fibrillary acidic protein, MBP: myelin basic protein.
