# Supplementary material for: Use of Ferritin Expression, Regulated by Neural Cell-Specific Promoters in Human Adipose Tissue-Derived Mesenchymal Stem Cells, to Monitor Differentiation with Magnetic Resonance Imaging In Vitro
Source: PLoS One. 2015 Jul 15;10(7):e0132480. doi: 10.1371/journal.pone.0132480 (PMC4503445; doi:10.1371/journal.pone.0132480)
Supplement: S2 Table — (DOC) [file pone.0132480.s002.doc]

**S2 Table. Neural differentiation procedures for neural-differentiation-inducible ferritin-expressing (NDIFE) human adipose tissue-derived mesenchymal stem cells (hADMSCs).**

| Differentiation into | Pre-induction (step 1) | | Induction (step 2) | | Evaluated marker |
| --- | --- | --- | --- | --- | --- |
|  | ingredients | duration | ingredients | duration |  |
| Neurons | Neurobasal medium  0.1 mM β-ME  10 ng/mL bFGF | 24 h | Neurobasal medium  10% FBS  3 mM β-ME | 48 h | NSE |
| Astrocytes | DMEM  2 mM L-glutamine  20 ng/mL EGF  20 ng/mL bFGF  1% N2 supplement | 72 h | DMEM  2 mM l-glutamine  1 mM dbcAMP  0.5 mM IBMX  5 ng/mL PDGF-AA  50 ng/mL NRG1  20 ng/mL bFGF | 72 h | GFAP |
| Oligodendrocytes | Neurobasal A medium  20 ng/mL bFGF  20 ng/mL EGF  1% N2 supplement  2 mM l-glutamine | 72 h | Neurobasal A medium  100 ng/mL IGF-1  50 ng/mL NT-3  5 ng/mL PDGF  1% N2 supplement | 72 h | MBP |

Sources of the reagents: Neurobasal medium, Neurobasal A medium, N2 supplement, l-glutamine, DMEM, β-ME, FBS (GIBCO, Grand Island, NY, USA); dbcAMP, IBMX (Sigma-Aldrich, Shanghai, China); bFGF, EGF, PDGF-AA, IGF-1, NT-3 (Peprotech, Rocky Hill, NJ, USA); NRG1 (R&D Systems, Inc., Minneapolis, MN, USA); rabbit anti-NSE antibody, mouse anti-GFAP antibody, mouse anti-MBP antibody (Abcam, Cambridge, MA, USA).

β-ME: 2-mercaptoethanol, FBS: fetal bovine serum, NSE: neuron-specific enolase, bFGF: basic fibroblast growth factor, DMEM: Dulbecco’s modified Eagle’s medium, GFAP: glial fibrillary acidic protein, EGF: epidermal growth factor, dbcAMP: N6,2′-O-dibutyryladenosine 3′,5′-cyclic monophosphate sodium salt, IBMX: 3-isobutyl-1-methylxanthine, PDGF-AA: platelet-derived growth factor AA, NRG1: neuregulin 1-β1/HRG1-β1 EGF domain, IGF-1: insulin-like growth factor 1, NT-3: neurotrophin-3, MBP: myelin basic protein.
