# Supplementary material for: Use of Ferritin Expression, Regulated by Neural Cell-Specific Promoters in Human Adipose Tissue-Derived Mesenchymal Stem Cells, to Monitor Differentiation with Magnetic Resonance Imaging In Vitro
Source: PLoS One. 2015 Jul 15;10(7):e0132480. doi: 10.1371/journal.pone.0132480 (PMC4503445; doi:10.1371/journal.pone.0132480)
Supplement: S3 Table — Table S3-1. Raw data of Cell Counting Kit-8 assay. Table S3-2. Raw data of Western blot. Table S3-3. Raw data of inductively coupled plasma mass spectrometry. Table S3-4. Raw data of MRI in vitro. (DOC) [file pone.0132480.s003.doc]

Raw data of “Use of Ferritin Expression, Regulated by Neural Cell-specific Promoters in Human Adipose Tissue-Derived Mesenchymal Stem Cells, to Monitor Differentiation with Magnetic Resonance Imaging In Vitro”

Table S3-1 (for Fig. 2. of main manuscript): Raw data of Cell Counting Kit-8 assay (Cell proliferation OD450nm)

| Cell proliferation (OD 450nm) | | Time (h) | | | |
| --- | --- | --- | --- | --- | --- |
|  |  | 24h | 48h | 72h | 96h |
| Group | Control | 0.898 | 1.260 | 1.375 | 1.467 |
|  |  | 0.840 | 1.211 | 1.424 | 1.388 |
|  |  | 0.880 | 1.242 | 1.411 | 1.420 |
|  | SYN1p-FTH1 | 0.865 | 1.218 | 1.426 | 1.388 |
|  |  | 0.944 | 1.241 | 1.397 | 1.448 |
|  |  | 0.905 | 1.275 | 1.462 | 1.418 |
|  | GFAPp-FTH1 | 0.867 | 1.273 | 1.467 | 1.474 |
|  |  | 0.943 | 1.235 | 1.409 | 1.467 |
|  |  | 0.911 | 1.202 | 1.472 | 1.388 |
|  | MBPp-FTH1 | 0.844 | 1.289 | 1.473 | 1.419 |
|  |  | 0.947 | 1.237 | 1.451 | 1.461 |
|  |  | 0.936 | 1.224 | 1.409 | 1.491 |

Table S3-2 (for Fig. 4A.of main manuscript): Raw data of Western blot

| Group | Relative ferritin protein expression level | | |
| --- | --- | --- | --- |
| SYN1p-FTH1 | 1.092 | 1.062 | 1.222 |
| GFAPp-FTH1 | 1.045 | 1.040 | 1.183 |
| MBPp-FTH1 | 0.978 | 1.005 | 1.173 |
| SYN1p-FTH1-N | 2.029 | 1.816 | 1.865 |
| GFAPp-FTH1-A | 1.952 | 1.968 | 2.131 |
| MBPp-FTH1-O | 2.051 | 1.817 | 1.850 |

Table S3-3 (for Fig. 5B. of main manuscript): Raw data of inductively coupled plasma mass spectrometry

| Group | Intracellular iron content (pg/cell) | | |
| --- | --- | --- | --- |
| SYN1p-FTH1 | 105.27 | 146.48 | 125.56 |
| GFAPp-FTH1 | 147.60 | 158.20 | 175.90 |
| MBPp-FTH1 | 137.49 | 107.18 | 109.00 |
| SYN1p-FTH1-N | 568.34 | 597.93 | 649.06 |
| GFAPp-FTH1-A | 617.85 | 638.79 | 669.31 |
| MBPp-FTH1-O | 544.84 | 613.78 | 633.31 |

Table S3-4 (for Fig. 6.of main manuscript): Raw data of MRI in vitro

| Group | R_2_ relaxation rate (S^-1^) | | | |
| --- | --- | --- | --- | --- |
| SYN1p-FTH1 | 9 | 8 | 9 | 8 |
| GFAPp-FTH1 | 10 | 9 | 9 | 9 |
| MBPp-FTH1 | 8 | 9 | 9 | 8 |
| SYN1p-FTH1-N | 29 | 27 | 31 | 27 |
| GFAPp-FTH1-A | 38 | 37 | 36 | 34 |
| MBPp-FTH1-O | 34 | 34 | 36 | 37 |
